# Advancing and critical appraisal of an integrative load monitoring approach in microcycles in professional soccer

**DOI:** 10.1371/journal.pone.0286372

**Published:** 2023-09-01

**Authors:** Linda Ammann, Ludwig Ruf, Adam Beavan, Paweł Chmura, Stefan Altmann

**Affiliations:** 1 TSG ResearchLab gGmbH, Zuzenhausen, Germany; 2 Department of Team Games, Wrocław University of Health and Sport Sciences, Wrocław, Poland; 3 Institute of Sports and Sports Science, Karlsruhe Institute of Technology, Karlsruhe, Germany; 4 Independent Researcher, Luzern, Switzerland; Instituto Politécnico de Santarém: Instituto Politecnico de Santarem, PORTUGAL

## Abstract

Despite load monitoring being considered an integral part of targeted performance management, including injury and illness prevention, there is currently no consensus of an effective monitoring system in professional soccer. Thus, the aims were to apply an integrative load monitoring approach, previously established in rink-hockey, in professional soccer; extend this approach with further data (Short Recovery and Stress Scale); assess this (extended) approach, thereby further evaluating the relationship between the used external load (EL) measures (total distance, distance above 55% and 70% of individual maximal speed, number of accelerations and decelerations > 4 m/s^2^, total loading) and the internal load (IL) measure session rate of perceived exertion training load (sRPE-TL) as well as between the used EL measures and sRPE. This retrospective observational cohort study analyzed data from a Swiss team collected over a 14 week-period during the 2021/22 season. Based on our findings, the integrative approach tested proved to be an applicable load monitoring tool in professional soccer, placing players on a fitness-fatigue continuum throughout the different microcycle sessions without using tests, thus providing relevant information to individually tailor training programs. sRPE-TL (ρ [95% CI] = .55 [.51 to .59] to .87 [.85 to .88]; all *p* < .001) better reflected the EL experienced by players than sRPE (ρ [95% CI] = .45 [.40 to .50] to .71 [.69 to .75]; all *p* < .001) supporting the definition of sRPE-TL as a measure of IL. However, for even stronger relevance of the tested tool, further research is warranted, especially to ascertain its sensitivity and determine an optimal selection of EL and IL measures. In sum, the present data clearly demonstrate the importance of load management taking place at an individual level, even within team structures, thereby analyzing a set of both EL and IL measures.

## Introduction

There is widespread agreement both in sport science and practice for load monitoring being an integral part of any targeted performance management, which also includes injury and illness prevention [1–5]. Training and competition load of athletes can be defined as input variable that coaches intending to elicit certain training induced adaptations (as this term including competitions) in their athletes usually try to manipulate [6]. Measures of training or competition load can be categorized as either external or internal, depending on whether they refer to measurable aspects occurring externally or internally to the athlete [2, 4, 5]. External loads (EL) are objective measures of the work performed by an athlete during training or competition. In contrast, internal loads (IL) refer to the relative biological (both physiological and psychological) stressors imposed on the athlete during training or competition [2, 4].

Within load monitoring, an integrated approach, rigorous and consistent, combining both external and internal loads, is considered to provide more significant information about the stress–which elicits various psychophysiological responses leading to training induced adaptations–experienced by athletes than interpretations based on isolated data [2, 4, 5]. The way that external and internal loads interact (i.e., on a continuum from coupling to divergence) may also allow to identify how athletes are coping with their training and to differentiate between a non-fatigued and a fatigued athlete [7] or further to identify the “state” of an athlete on a fitness-fatigue continuum [3, 4, 8]. In doing so, the term fitness is associated with the ability to cope with a certain level of standardized EL with a lower IL response than was recently the case. In contrast, a comparatively higher IL on a given EL under standardized conditions, up to the inability to complete a task that was once achievable within a recent time frame, is related to fatigue or a declining fitness level [3, 4, 7]. In the context of team sports, external and internal loads have been related through an internal-to-external or external-to-internal load ratio with the resulting quotient often termed efficiency index (Eff_index_) [8]. There are different ways to calculate the Eff_index_, and the most appropriate method needs to be determined in each team sport setting [8].

Despite and/ or due to a number of recent evolutions in the field of athlete load management, with various related factors and processes interacting [2, 3, 9, 10], there is currently no consensus of an effective monitoring system in professional soccer [2, 5, 10]. Specifically, relevant evolutions were: i) the relevance of professional load management could be established more widely [3, 5, 8, 11]; ii) new technologies nowadays offer numerous possibilities for continuous monitoring of various parameters of a player both inside and outside training sessions, which iii) has been accompanied by a massive increase in available (load) measures [2, 3, 5, 11]; iv) an increase in knowledge and a variety of different analytical approaches arising from enhanced research, including efforts to incorporate personal experiences and anecdotal information [2, 3, 5, 10]. However, it is evident, that there is also a need to ensure appropriate monitoring of individuals within a team environment [2, 3, 8, 10]. As it is recommended to practitioners to select or adapt evidence-based practices or analytical approaches situation-specific and based on a critical thinking process, with a healthy dose of skepticism and awareness of appropriate theoretical frameworks [2, 11, 12], the existence of different analytical load monitoring approaches seems reasonable.

**Fig 1:**
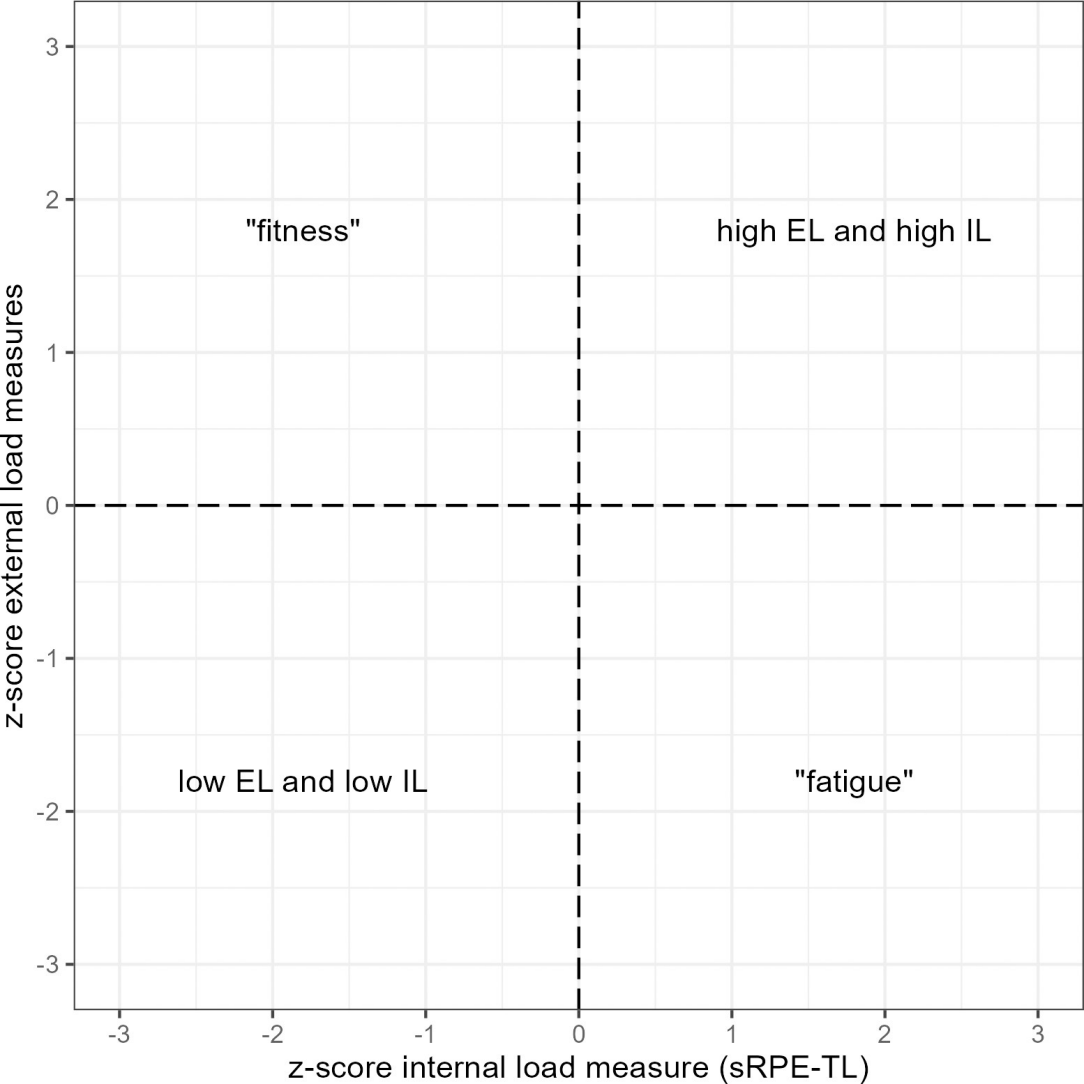
Fundamental principle of the descriptive method of the integrative load monitoring approach as used in the present study.

Recently, Fernández et al. (2021) [13] presented an integrative approach to external and internal load dynamics of rink hockey players for monitoring fitness and fatigue status of specific in-court training sessions in a standard one-match-per-week microcycle. By analyzing the relationship between EL and IL measures, the players are placed in the fitness-fatigue continuum throughout the different microcycle sessions without using tests. The information ultimately being presented in an easily understandable figure, which in its principle is shown in Fig 1, also means one of the important criteria for practical application can be met. This relates to a crucial aspect of effective monitoring that the large amount of data/ information can be simplified with reporting only a few key metrics and provide them to decision-makers in an easily accessible and engaging format [2, 3, 12].

Although the theoretical assumptions underlying the approach presented by Fernández et al. (2021) [13] appear to be coherent, this approach does not yet seem mature enough to be used in practice in a way that would undoubtedly add value. The most central point is, as far as now, it remains unclear whether indicated fatigue (resp. fitness) actually is fatigue (resp. fitness). If so, are meaningful differences detected? In addition, it is unclear what data (amount/ time span) is required to achieve significant information. Furthermore, to the best of our knowledge, there is no application of an approach similarly presented by Fernández et al. (2021) [13] in other sports than rink hockey. Especially in other sports, the selection and, if necessary, weighting of the external and internal load measures considered can possibly be optimized.

While a comparison with fatigue-related parameters may provide further insight into the fatigue-fitness question [14, 15], both theoretical-statistical and practice-contextual considerations seem to be necessary regarding data selection. As the Short Recovery and Stress Scale (SRSS) can provide information about the recovery-stress state of an athlete [16–18], extending the SRSS data to the approach presented by Fernández et al. (2021) [13] may yield further insights into how indicated fatigue (resp. fitness) signifies fatigue (resp. fitness).

The aim of this research is to: i) apply the integrative load monitoring approach presented in rink hockey by Fernández et al. (2021) [13] in professional soccer; ii) extend this approach with further data (Short Recovery and Stress Scale), iii) assess this (extended) approach by critically examining the classification of the player’s sessions on the fitness-fatigue continuum contextually and literature based, thereby further iv) evaluating the relationship between EL and IL (session rate of perceived exertion training load; sRPE-TL) measures as well as between EL measures and sRPE in professional soccer.

## Materials and methods

### Study design and research methods

A retrospective observational cohort study was implemented on a professional Swiss men soccer team over a 14 week-period during the second phase of the 2021–2022 season. In this phase the team competed in the highest national championship (Credit Suisse Super League®) as well as the national cup competition (Helvetia Schweizer Cup^TM^) in Switzerland. In addition to 15 championship matches and one cup match, there was one test match. Beside a one-week international break, in which the test match took place, ten weeks with one match and three weeks with two matches were scheduled.

The data analyzed came from daily routine monitoring of the players. Before each session multiple facets of recovery and stress states of an athlete were assessed using the SRSS. External and internal load data were collected during and after each training session and match, respectively. Data from rehabilitation, strength (no strength sessions at team level, possible through self-initiative) or additional off-court recreational training sessions were excluded. The same applies to on-field sessions which a player completed outside the team analyzed (e.g., U21). This study did not influence or alter the sessions in any way. The training sessions included all the activities on the pitch (i.e., warm-up, main part and, if performed, cool-down and/ or additional individual drills). For matches, only the match playing time was considered for both EL and IL (i.e., warm-up activities were excluded). The training sessions and matches took place on natural grass pitches and artificial turf pitches. The pitch surface in training was chosen depending on weather conditions, infrastructural conditions, and the next match.

With the aim of testing the approach presented by Fernández et al. (2021) [13] for daily applicability and with the idea that more data of an athlete may lead to more precise z-scores, the data of all sessions of the 14 week-period were considered for the analysis. This means, for example, regardless of whether at all respective how long a player was fielded in the previous or next match [19], the microcycle structure [20, 21] or situational and environmental conditions [22–24].

The training sessions were classified according to the number of days before or after a match day (i.e., matchday (md) minus or plus), with the assignment to a match being chosen according to the focus of a training session. For example, md-2 means a training session focused on upcoming match and took place 2 days before match day. In training sessions classified as matchday plus one (md+1), the players with little playing time in the preceding match trained on the pitch, while the players with more playing time followed an individual regeneration program off the pitch. The label general was assigned, if a training session was without focus on a match (e.g., first part of international break). If multiple sessions were scheduled at one day, EL and IL was summarized for each athlete.

Data (session of an athlete) were excluded from the analysis if no data was available for at least one of the external (*n* = 0) and internal (*n* = 260) load measures. Such missing values could be explained by problems with the devices used to measure the EL or by players not answering the sRPE-questionnaires. Furthermore, a minimum of 20 sessions per player was defined to have a certain basis for the calculation of z-score values, whereby the players featured a greater number of sessions for differently classified days of a microcycle and for which an associated build-up time (i.e., ca. 3 to 4 weeks) was considered reasonable. The total number of sessions included as well as the distribution of these sessions regarding their classification within a microcycle are summarized at the beginning of the results.

### Participants

A total of *N* = 27 field players of the team under observation were asked to take part in this study. Goalkeepers were excluded due to their different activity profile compared to field positions [25]. The eligible players were screened for health contraindications by the internal club sports medicine staff as part of their normal care of the team, which meant that the only inclusion criterion was participating in the team’s training and match activities during the data collection period. All of them gave written informed consent voluntarily. Although the data were collected as part of the players’ professional employment [26], ethical approval was obtained from a local ethics committee (Wrocław University of Health and Sport Sciences, Wrocław, Poland, identification number: 9/2023, 31 March 2023). After excluding players with less than 20 sessions with complete datasets (*n* = 2), the sample size considered for the analysis was *n* = 25. Selected biometric and positional data of these players are summarized at the beginning of the results.

### External load

A variety of external load measures were monitored for each player using global navigation satellite system (GNSS) technology (Apex Pro, STATSports, Newry, Ireland) (Apex Pro, STATSports, Newry, Ireland) with 10 Hz sampling. The validity and reliability of the STATSports Apex 10 Hz system were previously reported elsewhere [27–29]. Apex 10 Hz is a multi GNSS augmented unit, capable of acquiring and tracking multiple satellite systems (e.g., GPS, GLONASS, Galileo, BeiDou) concurrently to provide the best possible position information. The Apex GNSS model reports information about the number of satellites connected (*M* = 14.9, *SD* = 1.5, range 11 to 21), which was slightly lower than reported in previous literature [27, 28, 30]. The Apex units present the following characteristics: 30 mm (wide) × 80 mm (high) dimensions, 48 g weight, 100 Hz gyroscope, 100 Hz tri-axial accelerometer, and 10 Hz magnetometer. For each athlete an Apex unit was placed, according to manufacturer’s instructions, on the upper back between the right and left scapula through a vest. After data collection on the pitch, the Sonra software (Sonra 4.0, STATSports, Newry, Ireland) was used to download all data recorded by the GNSS and precisely define the session of each player (i.e., in trainings from the beginning of the official warm-up to the end of the last drill; in matches: the respective playing time). The data was then exported as a csv file for further analysis. To avoid inter-unit errors, players wore the same GNSS device and vest in each session.

The following external load measures were selected for analysis: total distance [m], distance above 55% of individual maximal speed [m], distance above 70% of individual maximal speed [m], number of accelerations > 4 m/s^2^, number of decelerations > 4 m/s^2^ and total loading [arbitrary units]. The distance-related measures, accelerations, decelerations, all of them with their respective thresholds, were selected because they have been used most frequently in practice and in studies analyzing external load (especially in soccer), and literature proposes to consider them [5, 10, 31, 32]. The latter also applies to total loading. Furthermore, these measures are similar to the one used by Fernández et al. (2021) [13]. Total loading gives the total of the forces on the player over the entire session based on accelerometer data alone and without any weightings. It uses the magnitude of the accelerometer values taken in three directions, sampled with 100 Hz. The total is scaled by 1000 to give manageable values. In many cases it is recommended to work with fixed speed thresholds [5, 10], which seems reasonable from various aspects. In contrast and given load management should take place at the individual level [2, 10], relative speed thresholds have been chosen for the purpose of the present analysis [31, 32]. The percentage thresholds applied for the relative speed thresholds (i.e., 55 and 70) are explained by the fact that they correspond to the recommended fixed thresholds [5] for a maximum speed of 36 km/h. In the present analysis, the individual maximum speed was defined as the respective highest speed measured by GNSS [33], provided it followed a proper acceleration phase, the absence of which reveals clear measurement errors. In case a new maximum speed was measured, the new value replaced the previous one.

### Internal load

The athletes were asked to complete a questionnaire after the end of each session (i.e., match or training), reporting a single global rating of their perceived exertion for the entire session (sRPE) [34] on a CR10-point scale, adapted by Foster et al. (2001) [34]. The questionnaire was to be filled in on the personal smartphone, ideally 30 minutes after a session. The players were instructed to provide the data honestly and individually. They were also told that no negative consequences could arise for them in connection with their answers. By multiplying declared sRPE by the respective session duration [min], originating from GNSS data, session rate of perceived exertion training load (sRPE-TL), presented in arbitrary units, was calculated to quantify internal load [5, 35, 36].

### Short Recovery and Stress Scale (SRSS)

The SRSS was used to assess multiple facets of recovery and stress states of an athlete. It proved to be a validated standardized psychometric tool among adults and adolescents [16, 17]. The SRSS is a derivation of the Acute Recovery and Stress Scale (ARSS) and as such contains eight items to be rated on a Likert-type scale ranging from 0 (“does not apply at all”) to 6 (“fully applies”) [16–18]. Each item is described by four adjectives, thus providing examples of each construct. Namely, the short recovery scale includes the constructs: physical performance, mental performance, emotional balance, and overall recovery; the short stress scale covers the constructs muscular strain, lack of activation, negative emotional state, and overall stress. The athletes were asked to fill in a questionnaire on their personal smartphones, ideally as a routine every morning after getting up, but at the latest one hour before the start of a session. They were familiarized with the questionnaire as well as told that their answers could not have any negative consequences for them. In this regard, they were explicitly instructed to provide their answers honestly and individually. It is worth noting that some players refused to answer the questionnaire for reasons unrelated to the study and beyond the researchers’ control, resulting in a data set of *n* = 968 available for analysis.

### Statistical analyses

All data were analyzed with the open-source software RStudio (R version 4.2.0 (2022-04-22 ucrt), R Core Team 2021, Wien, Austria [37]). Descriptive statistics were used to describe and characterize the sample. Thereby mean (SD) and range was reported.

A descriptive method was used to analyze the EL and IL measured in z-score values with regard to the number of days before or after the match. In doing so, a z-standardization was calculated for each external and internal load measure from the data basis, defined as described in detail above. This z-score calculation was performed grouped by athlete. After z-standardization, a mean value for the EL (EL mean) was calculated from the z-standardized EL variables (i.e., one EL value per athlete and session). A descriptive method was also used to gain further insight into the extent to which fatigue (resp. fitness) means fatigue (resp. fitness). As reporting habits differ between athletes (e.g., range of values; definition of the individual “normal” or “optimum” [18, 38]), this last part of the analysis was mainly carried out at an individual level.

Spearman rank correlations were employed to examine the relationship between the EL measures used and the IL measure sRPE-TL as well as between the EL measures used and sRPE (assumptions for Pearson product-moment correlation tests were judged not to be fulfilled). Spearman rank correlations were also calculated between the EL measures to assess whether there may be a weighting of any aspect in the calculated EL mean. In all three cases, alpha error accumulation was counteracted with a Bonferroni correction. In addition, a 95% confidence interval was calculated for the correlation coefficient *rho* (ρ). Furthermore, the correlation coefficient ρ was squared to get the determination coefficient *R*^*2*^. To be able to directly relate the results of the correlation tests to the analysis with the z-scores, the correlations are based on the same data basis (e.g., data summarized per day and athlete; athletes with < 20 sessions excluded). The magnitude of correlation coefficients was considered trivial (ρ < 0.1), small (0.1 < ρ < 0.3), moderate (0.3 < ρ < 0.5), large (0.5 < ρ < 0.7), very large (0.7 < ρ < 0.9), extremely large (0.9 < ρ < 1.0) and perfect (ρ = 1.0) [13, 36, 39]. The level of statistical significance was set to *p* < 0.05 for all tests.

## Results

A total of 1104 sessions with concurrent external and internal load data were identified for the sample and included in the analysis. The distribution of these sessions regarding their classification within a microcycle was: md-5 = 40, md-4 = 117, md-3 = 139, md-2 = 189, md-1 = 288, md = 174, and md+1 = 100. The mean number of sessions of an athlete was = 47.1 (*SD* = 10.4, range = 25 to 63). At the start of the data collection, the 25 professional soccer players participating in this study were on average 1.816 m in height (*SD* = 0.061 m, range = 1.66 to 1.95 m), and had an average body weight of 76.83 kg (*SD* = 7.32 kg, range = 64.0 to 89.4 kg). Their mean age was = 25.36 years (*SD* = 4.50 years, range = 18.5 to 36.5 years), and an average individual maximal speed of = 33.560 km/h (*SD* = 1.110 km/h, range = 31.93 to 36.32 km/h) was recorded. The distribution of the players by their field position was as follows: 4 central defenders, 4 full-backs, 11 midfielders, and 6 forwards.

**Fig 2:**
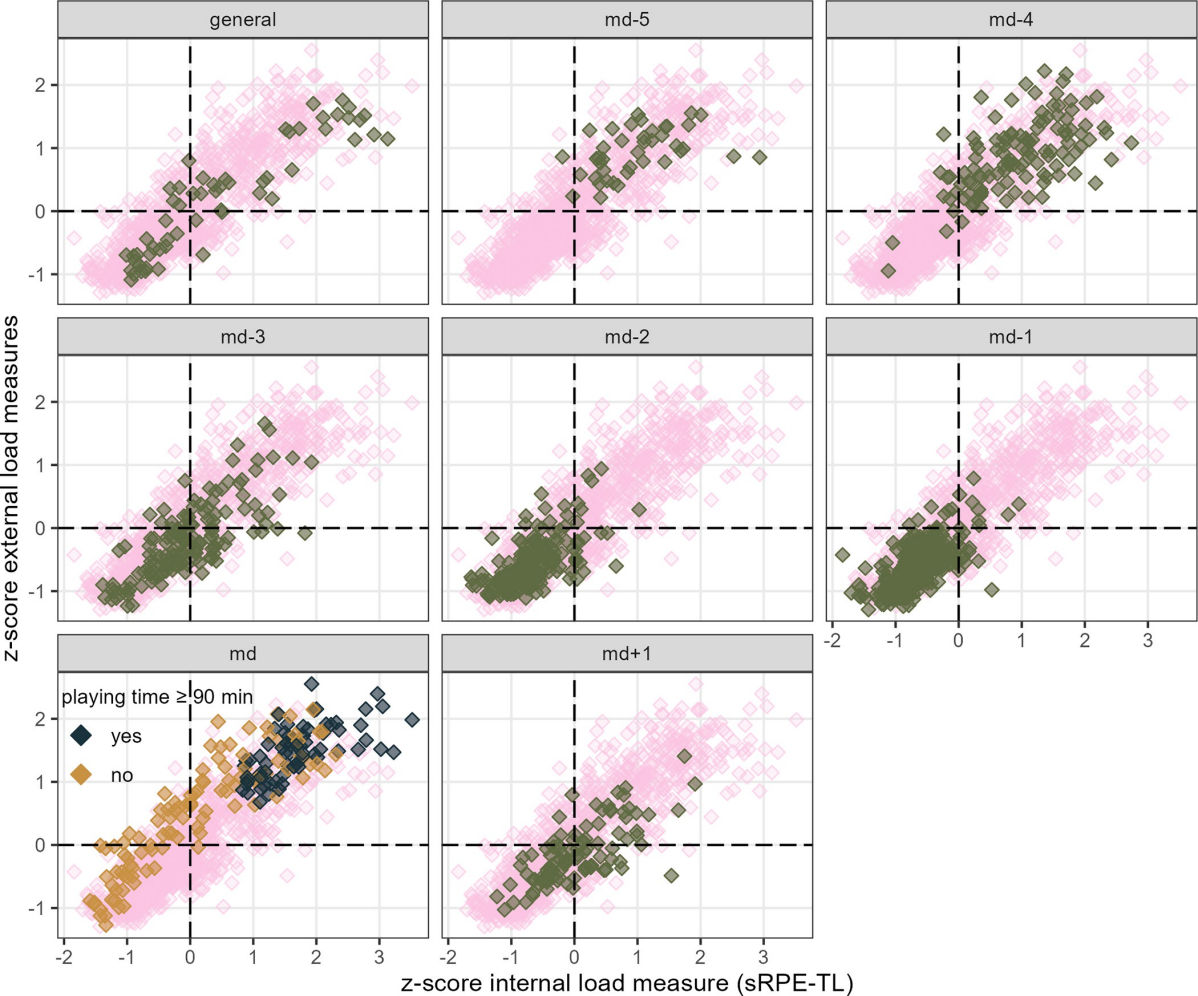
Distribution of the mean of the EL measures z-scores and the IL sRPE-TL z-scores of each athlete. The darker colored data points in the foreground show sessions classified regarding a microcycle as stated in the title of the respective facet, while the ghostly light pinkish data points in the background represent all sessions. As additional information, for matches it is indicated whether an athlete has played at least 90 minutes.

**Fig 3:**
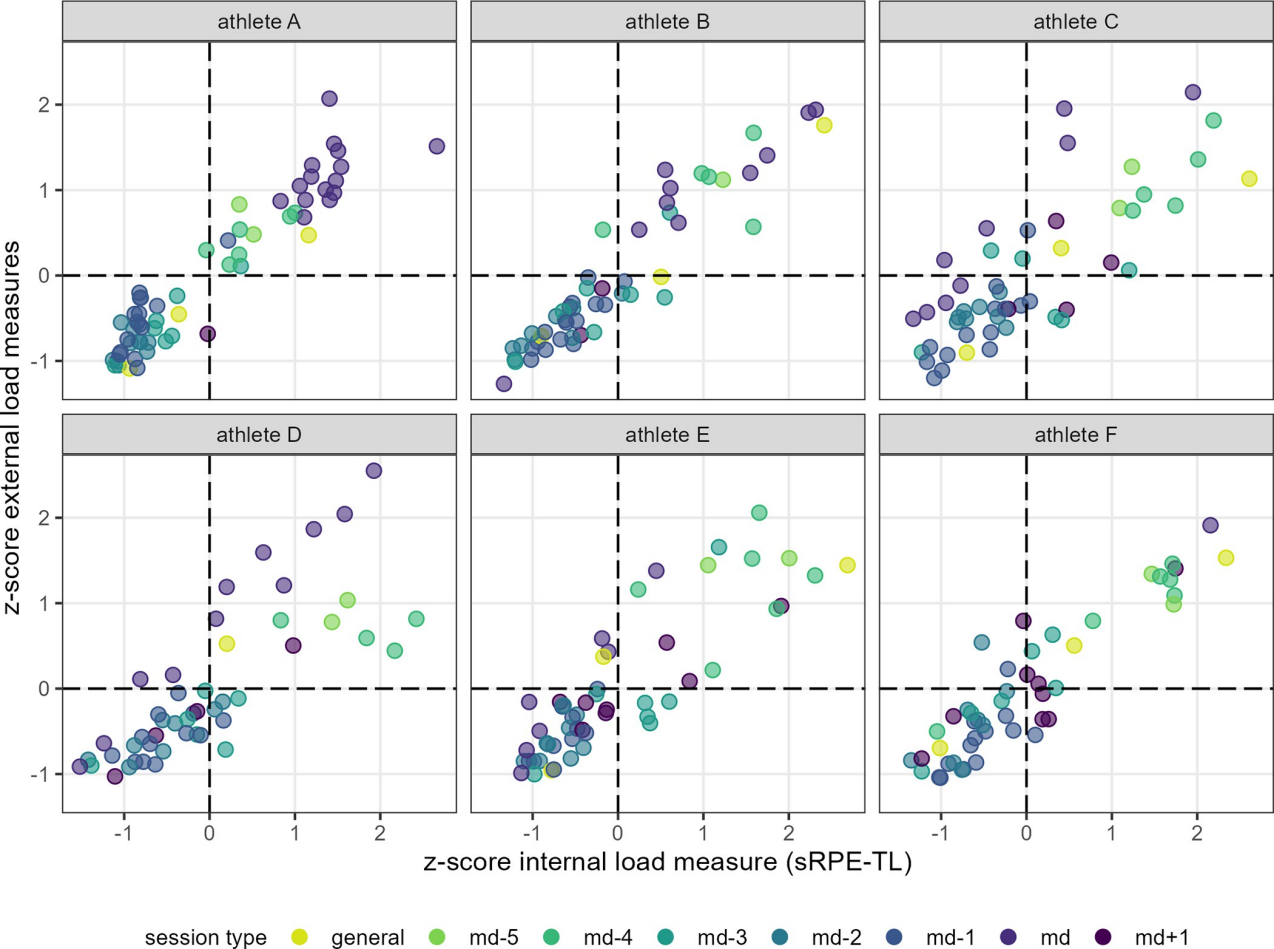
Distribution of the mean of the EL measures z-scores and the IL sRPE-TL z-scores of the one athlete of interest in each case. Session type shows the classification of the respective session in a microcycle. Athletes A, B: regular players; athletes C, D: players with regularly match activities; athletes E, F: players with only a few minutes of action in matches.

**Fig 4:**
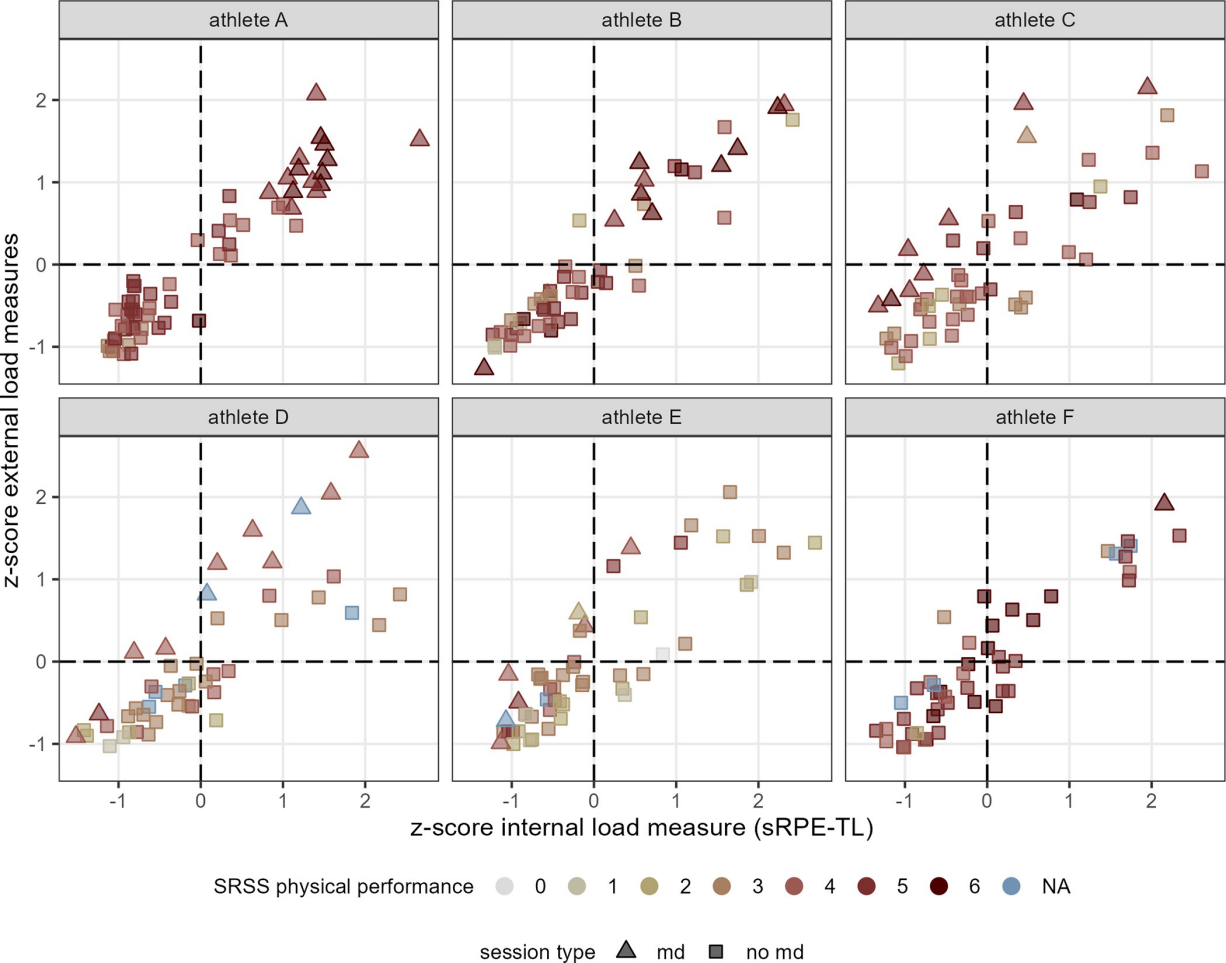
Distribution of the mean of the EL measures z-scores and the IL sRPE-TL z-scores of the one athlete of interest in each case with data points colored depending on an athlete’s SRSS item physical performance. 0 indicates an athlete judged the adjectives “powerful, able to perform, full of energy, full of power” as “does not apply at all”, whereby 6 indicates he rated them as “fully applies”. As additional information, the shape of the data points indicates whether the session was classified as matchday or not. NA: no SRSS data available. Athletes A, B: regular players; athletes C, D: players with regularly match activities; athletes E, F: players with only a few minutes of action in matches.

Fig 2 shows the application of the integrative approach presented by Fernández et al. (2021) [13], on team level, in professional soccer. Individual athlete’s data presented by Fig 3 indicate the general pattern of session placement observed in Fig 2 varies depending on a player’s match time. Identical data (athletes and session loads) are extended by athlete’s answers on the SRSS item physical performance (considered to be nearest item to the “state” of a player on a fitness-fatigue continuum) in Fig 4.

Table 1 presents the Spearman rank correlations between the selected EL measures and the IL measure sRPE-TL, while Table 2 presents the Spearman rank correlations between the selected EL measures and sRPE. There was an almost certainly large to almost certainly very large positive and thus stronger correlation with sRPE-TL (ρ = .55 to .87; all *p* < .001) than with sRPE (ρ = .45 to .71; all *p* < .001) for all EL measures, except for the distance > 70% of individual maximum speed, where the correlation with sRPE-TL (ρ = .55, *p* < .001) and sRPE (ρ = .56, *p* < .001) were nearly equal.

**Table 1:** Spearman rank correlation with Bonferroni correction between IL session rate of perceived exertion training load (sRPE-TL) and selected external load (EL) measures.

EL measure	ρ [95% CI]	*p*	*n*	*R* ^*2*^
Accelerations > 4 m/s^2^	.59 [.54 to .62]	< .001*	1104	.34
Decelerations > 4 m/s^2^	.70 [.67 to .73]	< .001*	1104	.49
Distance > 55% of individual maximal speed	.69 [.66 to .72]	< .001*	1104	.48
Distance > 70% of individual maximal speed	.55 [.51 to .59]	< .001*	1104	.30
Total distance	.87 [.85 to .88]	< .001*	1104	.75
Total loading	.84 [.83 to .86]	< .001*	1104	.71

Presented are correlation coefficient ρ with 95% confidence interval, p value, sample size n as well as determination coefficient *R*^*2*^. Level of significance

* *p* < 0.05.

**Table 2:** Spearman rank correlation with Bonferroni correction between session rate of perceived exertion (sRPE) and selected external load (EL) measures.

EL measure	ρ [95% CI]	*p*	*n*	*R* ^*2*^
Accelerations > 4 m/s^2^	.45 [.40 to .50]	< .001*	1104	.21
Decelerations > 4 m/s^2^	.66 [.62 to .69]	< .001*	1104	.43
Distance > 55% of individual maximal speed	.66 [.62 to .69]	< .001*	1104	.43
Distance > 70% of individual maximal speed	.56 [.52 to .60]	< .001*	1104	.31
Total distance	.72 [.69 to .75]	< .001*	1104	.52
Total loading	.70 [.67 to .73]	< .001*	1104	.49

Presented are correlation coefficient ρ with 95% confidence interval, p value, sample size n as well as determination coefficient *R*^*2*^. Level of significance

* *p* < 0.05.

**Fig 5:**
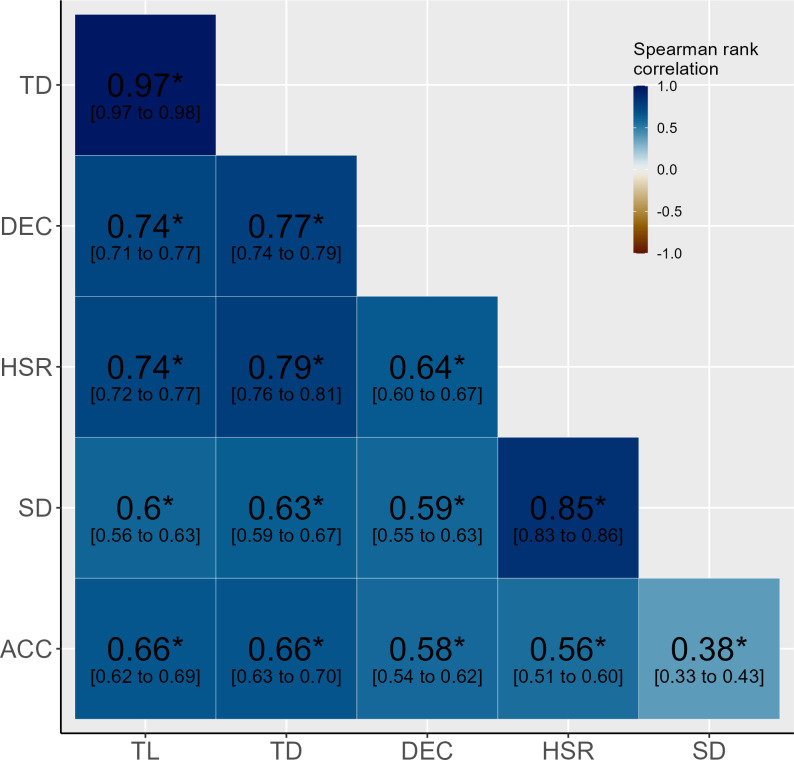
Spearman rank correlation with Bonferroni correction between the selected external load measures. Presented are the correlation coefficient ρ with 95% confidence interval. Level of significance: * *p* < 0.05. Sample size *n* = 1127 for all comparisons. ACC = number of accelerations > 4 m/s^2^; DEC = number of decelerations > 4 m/s^2^; HSR (high speed running) = distance above 55% of individual maximal speed [m]; SD (sprint distance) = distance above 70% of individual maximal speed [m]; TD = total distance [m]; TL = total loading [arbitrary units].

Fig 5 displays the Spearman rank correlations between the selected EL measures.

## Discussion

Figs 2 and 3 show the application of the integrative approach presented by Fernández et al. (2021) [13] in professional soccer, i.e., the external and internal load measures are merged into an integrated system using z-scores, leading to a monitoring tool placing the players in a fitness-fatigue continuum throughout the different microcycle sessions without using tests. Here, we consider important to emphasize, that the placement of a session of a player in a fitness-fatigue continuum always shows the load of a specific session in dependence of all player-specific experienced session loads and thus is based on the underlying player-specific data. Hence, when working with this approach, the mistake should not be made to assume equal EL and IL z-score values between athletes correspond to the same absolute values of EL and IL experienced by the athletes.

### Session placement

On days in a microcycle further ahead of an upcoming match (md-5, md-4), players were placed in a “high EL and high IL” zone with only a few exceptions as shown in Fig 2. I.e., the EL the players were exposed to was high in most cases and they experienced correspondingly high IL. The observed exposure to more intense stimuli at a greater distance from a match is in accordance with previous research [10, 21, 40, 41]. Similar to md-5 and md-4, players on md-2 and md-1 showed an expected reaction (low IL) to the load experienced (low EL) in most cases. While the distribution of the sessions was at its lowest on these two days, directly preceding a match; md, md-3 and md+1 showed the largest dispersion as well as the range of EL experienced by the players was the greatest. In addition, md, md-3 and md+1 sessions were more often related to a fatigued state of the players, or else the players performed them more frequently in a state indicating fitness. An increased association with fatigue on md-3 and md+1 seems reasonable considering players were often exposed to higher loads the day before. The lowest external and internal loads measured in z-score values in the population analyzed on md-2 and md-1 for the whole team as well as on md+1 for the players with a lot of playing time suggest an intended recovery focus. The load reduction observed at the whole team level on the two days preceding a match is in line with what is known for soccer [10, 21, 40, 41] and may very roughly be located in the context of tapering, i.e., aiming for the best possible performance readiness [2, 42, 43]. Matches in which the players played for 90 minutes were among the highest load levels experienced in a microcycle by the respective players, an observation in line with the existing literature [21, 44]. In our analysis, no match session was associated with fatigue. This observation, as well as matches in which players did not play over the full duration increasingly associated with a state indicating fitness, may be explained by players reaching best possible readiness on matchday (meaning the coaches plans worked out), as well as contextual stimulating factors leading to comparatively lower perceived effort [35]. The placement of the md+1 sessions is also reported from other soccer populations [10, 45] and correspond to the loading design intended by the coaching staff of the present observation (personal communication), i.e., loading players depending on their playing time on the day before. Players with more playing time should be allowed to recover as quickly and optimally as possible, while those with less playing time should be exposed to a certain amount of load.

The breakdown of Fig 2 at an individual level (i.e., Fig 3) shows how the placement of sessions, classified in dependence of their temporal distance to a match, is related to the nature of match activities of a respective player. While regular players show a pattern which is roughly shown in Fig 2 and was presented in the same way by Fernández et al. (2021) [13], other days in the microcycle are increasingly associated with higher load for an athlete as match playing time decreases. This observation, along with Fig 4, showing reporting habits differing between athletes (e.g., range of values; definition of the individual “normal” or “optimum” [18, 38]), clearly highlights the importance of individual load management even within team structures as well as the importance of interpreting such data at individual rather than team level.

Under the aspects discussed so far, the classification of the sessions on the fitness-fatigue continuum seems reasonable. The extension of the integral approach by the SRSS data (Fig 4) does not contradict this in any way. It is important to note that, as mentioned above, players with more playing time in a match were exposed to a lower EL up to were absent in pitch-sessions on the subsequent md+1 in the present observation, explaining “missing” sessions with assumed low SRSS physical performance values in Fig 4. Furthermore, the lower physical performance capacity reported by the players being associated with training sessions in which the players were exposed to low EL (especially athlete B, C and D in Fig 4) appears plausible both in view of the load distribution over a microcycle as well as considering higher loads may induce fatigue on the subsequent day and is then recognizable in the players’ SRSS responses. However, considering i) the few data points related to ii) tendential fatigued respective fit states of players in the present soccer observation, further research is required to gain deeper insights into the sensitivity of this integral approach. Possibly, also analysis performed over other time periods (e.g., including preparation or off-season training programs [46, 47]) may yield further insights regarding the sensitivity of this integral approach. Such studies may further indicate whether, given chronic adaptations in physical fitness, it is required to exclude certain older data from the z-score calculations (to continue) to detect shorter-term “states” of players in a fitness-fatigue continuum. Thereby also further fitness and fatigue associated measures and tests (e.g., creatine kinase, lactate concentration, tests to assess cardiorespiratory fitness, jump protocols [5, 7, 46]) may reveal deeper insights. Despite this lack of certainty and thus concluding up to this point: the integrative approach applied seems to provide practitioners in professional soccer with relevant information to individually tailor training programs.

### Assessed measures

The observed correlations ranged from almost certainly large to almost certainly very large positive and thus were stronger with sRPE-TL than with sRPE (almost certainly moderate to very likely very large positive) for all EL measures, except for the distance > 70% of individual maximum speed, implying sRPE-TL better reflects the EL experienced by players than sRPE. This supports the definition and thus the use of sRPE-TL—and explicitly not sRPE—as an internal load measure [5, 10, 36]. Furthermore, and as reported in the same way from rink-hockey by Fernández et al. (2021) [13], the observed correlations suggest not only EL variables to affect IL response, but also other factors (individual characteristics, sociological factors, psychological status, health, nutrition, environment, etc. [4, 35]). Accelerations excluded, the lowest correlation observed between two selected EL measures was almost certainly large, indicating all of these EL measures represent parts of the complexity of soccer. The nevertheless varying degree of association between EL measures indicates they provide complementary information. Taken together, these findings clearly underline the need to assess i) a set of ii) both EL and IL measures.

The almost certainly extremely large positive correlation between total distance and total loading implies the total distance received a higher weighting in the calculated EL mean of the integrative approach analyzed. This might be judged appropriate considering total distance, in line with the literature [36], showing the strongest association with the IL sRPE-TL. In the present analysis, we employed threshold-based counts for accelerations, as it is common in various studies and recommended in the literature [5, 48, 49]. However, Sonderegger et al. (2016) [50] showed that if the running speed immediately prior to an acceleration being initiated and the maximal acceleration capacity associated with it are not considered, a number of high-intensity accelerations could be missed. I.e., arbitrarily set thresholds lead to accelerations from low speeds being overestimated and accelerations from high speeds being underestimated. We assume this to be the reason for the comparatively low relationship observed of accelerations with sRPE, with sRPE-TL as well as with the other EL measures and thus recommend (to test) the definition of accelerations according to Sonderegger et al. (2016) [50] in further studies.

Moreover, further research should evaluate possible optimization of this integrative approach by assessing other (combinations of) EL and IL measures included. Also, as applied for EL, the calculation of an IL mean may be worth investigating. Especially, if athletes are not ideally familiarized and trained in the appropriate method for providing sRPE, an integration of other (more objective) IL measures suggest making this integrative approach more robust–by being less affected trough not at least e.g., athletes showing social desirability or athletes trying to falsely influence subsequent training sessions [2, 35]. Unfortunately, and contrary to initial intention, it was not possible to record heart rate values in matches, making it impossible to include heartrate-based IL measures (e.g., Edwards trainings load) into this present analysis. It was beyond the scope of this study to derive training (i.e., the exposure to EL and the experience of IL) recommendations; thus, sport scientists and practitioners are encouraged to investigate the interactions of training interventions and the placement of sessions.

### Conclusion / practical application

The present data underline the importance of load management in professional soccer taking place at an individual level, even within team structures, thereby analyzing a set of both external and internal load measures. Based on our findings, we conclude the integrative approach analyzed in this work provides valuable information for this purpose. However, for even stronger relevance of the tool, further research is required particularly to ascertain its sensitivity and determine an optimal selection of included external and internal load measures. Furthermore, sRPE-TL better reflecting the external load experienced by players than sRPE supports the definition and thus the use of sRPE-TL–and explicitly not sRPE–as a measure of internal load.
